# A novel method for identifying geomechanical parameters of rock masses based on a PSO and improved GPR hybrid algorithm

**DOI:** 10.1038/s41598-022-09947-7

**Published:** 2022-04-05

**Authors:** Hanghang Yan, Kaiyun Liu, Chong Xu, Wenbo Zheng

**Affiliations:** 1 School of Civil Engineering, Beijing Jiaotong University, Beijing, 100044 China; 2 China Railway First Survey and Design Institute Group Ltd., Xi’an, 710043 Shanxi China; 3 School of Engineering, University of Northern British Columbia, Prince George, BC V2N 4Z9 Canada

**Keywords:** Civil engineering, Computer science

## Abstract

In view of the shortcomings of existing artificial neural network (ANN) and support vector regression (SVR) in the application of three-dimensional displacement back analysis, Gaussian process regression (GPR) algorithm is introduced to make up for the shortcomings of existing intelligent inversion methods. In order to improve the generality of the standard GPR algorithm with single kernel function, an improved Gaussian process regression (IGPR) algorithm with combined kernel function is proposed by adding two single kernel functions. In addition, in the training process of IGPR model, the particle swarm optimization (PSO) is combined with the IGPR model (PSO-IGPR) to optimize the parameters of the IGPR model. After the IGPR model can accurately map the relationship between geomechanical parameters and rock mass deformation, the PSO algorithm is directly used to search the best geomechanical parameters to match the deformation calculated by igpr model with the measured deformation of rock mass. The application case of Beikou tunnel shows that the combined kernel function GPR has higher identification accuracy than the single kernel function GPR and SVR model, the IGPR model with automatic correlation determination (ARD) kernel function can obtain higher identification accuracy than the IGPR model with isotropic (ISO) kernel function, and the PSO-IGPR hybrid model based on ARD kernel function has the highest identification accuracy. Therefore, this paper proposes a displacement back analysis method of the PSO-IGPR hybrid algorithm based on ARD kernel function, which can be used to identify the geomechanical parameters of rock mass and solve other engineering problems.

## Introduction

Back analysis techniques are often used in geotechnical engineering to determine unknown geomechanical parameters of rock masses by using field measurements of displacements, strains, and stresses during excavation or construction phases^1–5^. The displacement of rock masses induced by excavation can be measured reliably with relative ease. Therefore, displacement back analysis is the most common and effective method for identifying geomechanical parameters in rock engineering^6–13^. There are two types of displacement back analysis, namely, the inverse solving method and the direct optimization method. Compared with the inverse solving method, the direct optimization inversion method is not limited by the constitutive relationship of materials and has better applicability.

Two-dimensional numerical analysis is typically used in displacement back analysis to shorten the calculation time to meet the needs of engineering construction. However, in many cases, two-dimensional numerical simulations cannot account for the time and spatial effects of engineering construction. Several studies have introduced three-dimensional (3D) numerical simulations into displacement back analysis^12–14^, however, the computation time of 3D displacement back analysis is costly. Moreover, the parameters of rock masses exhibit considerable uncertainty, variability, and nonlinearity. Based on the advantages of fast calculation, self-fault tolerance, self-adaptation and considering high uncertainty, artificial intelligence technology is also widely used in the field of geotechnical engineering^15–20^.Thus, the intelligent back analysis method was introduced by incorporating artificial intelligence (AI) into 3D displacement back analysis because of AI’s high efficiency, good convergence, self-fault tolerance, and self-adaptation. In this method, a trained intelligent learning machine instead of 3D numerical simulations is used to conduct displacement back analysis, which is useful in identifying parameters for geotechnical engineering studies.

The performance of the learning machine is critical for achieving intelligent displacement back analysis. Previous intelligent back analyses have mainly used artificial neural networks (ANNs) as learning machines^21–26^. However, based on the empirical risk minimization principle, the generalization performance of an ANN as a learning machine works well only when the sample size is massive. In addition, ANNs have defects related to local optimization and overfitting. A support vector machine (SVM) can compensate for nearly all of the defects of ANNs because it is built on the structural risk minimization principle. An SVM-based algorithm, support vector regression (SVR), has been used for displacement back analysis and can obtain better application results than those with ANNs^27–34^. However, there are still two problems associated with the SVR displacement back analysis method. First, the estimated output of SVR is not probabilistic. Second, SVR is not highly effective when the version space is elongated or asymmetrical^35^.

As the Gaussian process (GP) has good generalizability and is highly suitable for solving high-dimensional, small-sample, and nonlinear classification and regression problems. Compared with ANNs and SVMs, the GP is easy to implement without sacrificing performance, and its hyperparameters can be obtained by calculating the maximum value of the log-likelihood function of training samples. In addition, the GP is a probabilistic kernel learning machine that has flexible nonparametric inference and probabilistic interpretation of the predicted output^35,36^. GPs have been successfully applied in geotechnical engineering^36–38^ and other fields^39^. However, the generalization performance of the GP with a single kernel function has certain limitations. For example, the classical method for determining optimal hyperparameters in the GP is the conjugate gradient method; however, this method has a strong dependence on the initial iteration value and the number of iterations and can result in local optima. These defects have primarily limited the application of GPs in the field of displacement back analysis.

Rasmussen and Williams proposed a combination kernel function to improve the generalizability of GP with a single kernel function^40^. In addition, many scholars have shown that the frequency distributions of rock strength can be well represented by a normal distribution^41–49^. In this paper, we apply this improved Gaussian process algorithm (IGPR) with a combination kernel function as a substitute for the standard GP with a single kernel function. Furthermore, Some optimization algorithms are used to search the optimal parameters of the learning machine to improve the generalization ability of the learning machine^50,51^.A particle swarm optimization (PSO) algorithm is proposed to replace the conjugate gradient method to determine the optimal hyperparameters of the GP in view of the above problems of the conjugate gradient method. To determine the geomechanical parameters for rock masses, the PSO and IGPR (PSO-IGPR) hybrid algorithm is introduced to the field of displacement back analysis combined with construction monitoring of the Beikou tunnel on the Zhangjiakou-to-Shijiazhuang Expressway in Hebei Province, China. To demonstrate the performance of the improved intelligent displacement back analysis, the results of using a PSO and SVR (PSO-SVR) hybrid algorithm and a PSO and standard GPR (PSO**-**GPR) hybrid algorithm with a different single kernel function are analysed and compared.

The layout of this work is organized as follows. The standard GPR and the IGPR algorithm are described in Section “Improved Gaussian process regression (IGPR) algorithms”. Section “PSO-IGPR hybrid algorithm for geomechanical parameter identification” introduces the method for theoretically identifying the geomechanical parameters of rock masses based on the PSO-IGPR hybrid algorithm. In Section “Case study on Beikou tunnel”, the PSO-IGPR hybrid algorithm is used to identify the geomechanical parameters of rock masses around the Beikou tunnel, which verifies the validity and accuracy of the novel displacement back analysis method proposed in this paper. Finally, concluding remarks are given in Section “Conclusions”.

## Improved Gaussian process regression (IGPR) algorithms

The GP is a type of machine learning method based on the Gaussian stochastic process and Bayesian learning theory. In the theory of statistics, the GP is a random process, and the distribution of any finite set of variables is a Gaussian distribution. That is, the joint probability distribution of any set of random variables {***x***_*i*_ ∈ ***X***, *i* = 1, …, *n*} and its corresponding process state {*Y*(***x***_1_), …, *Y*(***x***_*n*_)} follows an *n*-dimensional Gaussian distribution. All statistical characteristics of a GP are determined by its mean value and covariance functions^35,40^

1 $$\left. \begin{gathered} \mu ({\varvec{x}}) = E[Y({\varvec{x}})] \hfill \\ C({\varvec{x}},{\varvec{x}}^{^{\prime}} ) = E[(Y({\varvec{x}}) - \mu ({\varvec{x}}))(Y({\varvec{x}}^{^{\prime}} ) - \mu ({\varvec{x}}^{^{\prime}} ))] \hfill \\ \end{gathered} \right\}$$

where ***x***, ***x***^′^ ∈ ***X*** are both arbitrary random variables and ${\mathbf{x}}_{i}$ is a multi-dimensional input vector. The GP can be defined as $f({\varvec{x}})\sim GP(\mu ({\varvec{x}}),C({\varvec{x}},{\varvec{x}}^{^{\prime}} ))$.

A set of *n* observation data ${\varvec{D}} = \{ ({\varvec{x}}_{i} ,t_{i} ),i = 1, \cdots ,n\}$ is considered the training set of the Gaussian model. The observed target ***t*** is corrupted by noise, and the difference in the actual output value is *ε*; thus, the Gaussian noise model can be written as follows:

2 $$t_{i} = f({\varvec{x}}_{i} ) + \varepsilon_{i} ,i = 1,...,n\;\;\;\;$$

where *t*_*i*_ denotes the output scalar, *ε* denotes independent random variables with $\varepsilon \sim N(0,\sigma_{n}^{2} )$ (N is the normal distribution) and $\sigma_{n}$ denotes the variance of the noise. Under the framework of Bayesian linear regression *f*(*x*) = *Ψ*(*x*)^T^*w*, the prior distribution of the target value *t* of Eq. (1) is obtained as:

3 $$\left. \begin{gathered} {\varvec{Q}} = {\varvec{C}} + \sigma_{n}^{2} {\varvec{I}} \hfill \\ {\varvec{t}}\sim N(0,{\varvec{Q}}) \hfill \\ \end{gathered} \right\}$$

where ***I*** denotes the unit matrix. According to Eq. (3), the joint Gaussian prior distribution of the training sample output ***t*** and a test sample output t* can be deduced as follows:

4 $$\left\{ \begin{gathered} {\varvec{t}} \hfill \\ {\varvec{t}}^{*} \hfill \\ \end{gathered} \right\} \sim N\left( {\begin{array}{*{20}c} {0,} & {\left[ {\begin{array}{*{20}c} {{\mathbf{C}}({\mathbf{X}},{\mathbf{X}}) + \sigma_{n}^{2} {\mathbf{I}}} & {{\mathbf{C}}({\mathbf{X}},{\mathbf{x}}^{*} )} \\ {{\mathbf{C}}({\mathbf{x}}^{*} ,{\mathbf{X}})} & {{\mathbf{C}}({\mathbf{x}}^{*} ,{\mathbf{x}}^{*} )} \\ \end{array} } \right]} \\ \end{array} \, } \right)$$

where ${\mathbf{C}}({\mathbf{X}},{\mathbf{X}})$ is a $n \times n$ positive definite covariance matrix. Its arbitrary $c_{ij}$ measures the correlation between ${\mathbf{x}}_{i}$ and ${\mathbf{x}}_{j}$. ${\mathbf{C}}({\mathbf{X}},{\mathbf{x}}^{*} )$ is a $n \times 1$ covariance matrix of the tested ${\varvec{x}}^{*}$ and the input points of ***X***, and ${\mathbf{C}}({\mathbf{x}}^{*} ,{\mathbf{x}}^{*} )$ denotes the covariance of test point ${\mathbf{x}}^{*}$.

For a given test point ${\mathbf{x}}^{*}$ and training set ***D***, the goal of Bayesian probability prediction is to calculate $p({\varvec{t}}^{*} \left| {{\mathbf{x}}^{*} ,{\varvec{D}}} \right.)$. According to Bayesian posterior probability, Eq. (5) can be obtained:

5 $$p({\varvec{t}}^{*} {|}{\mathbf{x}}^{*} ,{\mathbf{D}}) \sim N(\mu_{{{\varvec{t}}^{*} }} ,\sigma_{{{\varvec{t}}^{*} }}^{2} )$$

The expectation and variance of ${\varvec{t}}^{*}$ are as expressed by Williams^35^:

6 $$\mu_{{{\varvec{t}}^{*} }} = {\mathbf{C}}({\mathbf{x}}^{*} ,{\mathbf{X}})({\mathbf{C}}({\mathbf{X}},{\mathbf{X}}) + \sigma_{n}^{2} {\mathbf{I}})^{ - 1} {\varvec{t}}$$

7 $$\sigma_{{t^{*} }}^{2} = {\varvec{C}}({\varvec{x}}^{*} ,{\varvec{x}}^{*} ) - {\varvec{C}}^{T} ({\varvec{x}}^{*} ,{\varvec{X}})({\varvec{C}}({\varvec{X}},{\varvec{X}}) + \sigma_{n}^{2} {\varvec{I}})^{ - 1} {\varvec{C}}({\varvec{x}}^{*} ,{\varvec{X}})$$

Because the covariance function of the GP method in the finite set of input requirements, which is a symmetric function satisfying the Mercer condition, is positive, the covariance function is equivalent to the kernel function. Equations (6) and (7) can be rewritten as:

8 $$\mu_{{{\varvec{t}}^{*} }} = K({\mathbf{x}}^{*} ,{\mathbf{X}})(K({\mathbf{X}},{\mathbf{X}}) + \sigma_{n}^{2} {\mathbf{I}})^{ - 1} {\varvec{t}}$$

9 $$\sigma_{{t^{*} }}^{2} = K({\varvec{x}}^{*} ,{\varvec{x}}^{*} ) - K^{T} ({\varvec{x}}^{*} ,{\varvec{X}})(K({\varvec{X}},{\varvec{X}}) + \sigma_{n}^{2} {\varvec{I}})^{ - 1} K({\varvec{x}}^{*} ,{\varvec{X}})$$

The mean value of the prediction is a linear combination of kernel function ***K****.* The nonlinear relational data can be mapped into the feature space and transformed into a linear relation; therefore, the complex nonlinear problem can be transformed into a linear problem.

Kernel functions can be divided into two categories: isotropic (ISO) kernel functions and automatic relevance determination (ARD) kernel functions. In this paper, the following two types of ARD kernel functions are applied:

1. Square exponential (SE) kernel function
  10 $$K_{SE} ({\varvec{x}}_{i} ,{\varvec{x}}_{j} ) = \sigma_{f}^{2} \exp \left[ { - \frac{{({\varvec{x}}_{i} - {\varvec{x}}_{j} )^{T} M({\varvec{x}}_{i} - {\varvec{x}}_{j} )}}{2}} \right] + \sigma_{n}^{2} \delta_{ij}$$
2. Rational quadratic (RQ) kernel function
  11 $$K_{RQ} ({\varvec{x}}_{i} ,{\varvec{x}}_{j} ) = \sigma_{f}^{2} \left[ {1 + \frac{{({\varvec{x}}_{i} - {\varvec{x}}_{j} )^{T} M({\varvec{x}}_{i} - {\varvec{x}}_{j} )}}{2\alpha }} \right]^{ - \alpha } + \sigma_{n}^{2} \delta_{ij}$$
  where $\left\{ M \right\} = diag({\mathbf{\ell }}^{ - 2} )$ denotes the diagonal matrix of the hyperparameters and ${\mathbf{\ell }}$ represents the hyperparameter, which determines the relevance between the input variable and the output variable. A larger value of ${\mathbf{\ell }}$ indicates a lower degree of relevance between the input and output variables. In addition, $\sigma_{f}^{2}$ denotes the signal variance of the kernel function and can be used to control the degree of local relevance, $\alpha$ represents the shape parameter of the kernel function, $\sigma_{n}^{2}$ is the noise variance, and $\delta_{ij}$ is the Kronecker symbol:
  12 $$\delta_{ij} = \left\{ {\begin{array}{*{20}c} {1,i = j} \\ {0,i \ne j} \\ \end{array} } \right.$$

The ARD-type kernel function and the ISO-type kernel function are the same in form; the difference between the two types of functions is the dimension of hyperparameter ${\mathbf{\ell }}$. The dimension of ${\mathbf{\ell }}$ is the same as that of the input variable ***x*** in the ARD-type kernel function. In other words, if the input variable ***x*** is a *d*-dimensional vector, ${\mathbf{\ell }}$ is also a *d*-dimensional vector. However, for the ISO-type kernel function, the dimension of ${\mathbf{\ell }}$ cannot be changed with the input variable dimension. Instead, it is always a one-dimensional scalar, which means that the relevance between all components of the input variable ***x*** and the output variable is the same^35,40^.

The hyperparameters of the kernel function have a considerable influence on the learning and prediction results. In the GPR algorithm, the optimal hyperparameters can be adaptively obtained by using the maximum likelihood method to establish a log marginal likelihood function of training samples and then obtaining the partial derivative of the hyperparameters. Finally, the optimum solution of the hyperparameters can be calculated using the conjugate gradient optimization method as follows^35,40^:

13 $$L = \ln [p({\mathbf{t}}|{\mathbf{X}},{\varvec{\theta}})] = - \frac{1}{2}{\mathbf{t}}^{{\text{T}}} {\mathbf{Q}}^{ - 1} {\mathbf{t}} - \frac{1}{2}{\text{ln|}}{\mathbf{Q}}{|} - \frac{n}{2}\ln (2{\uppi )}$$

14 $$\frac{\partial }{{\partial \theta_{i} }}\ln [p({\mathbf{t}}|{\mathbf{X}},{\varvec{\theta}})] = \frac{1}{2}{\text{tr}}\left[ {(\user2{\alpha \alpha }^{{\text{T}}} - {\varvec{Q}}^{ - 1} )\frac{{\partial {\varvec{Q}}}}{{\partial \theta_{i} }}} \right]$$

where ${\varvec{\theta}}$ denotes the vector that contains all of the hyperparameters.

The following combination kernel (CK) function can be obtained by adding Eqs. (10) and (11) to improve the generalizability of GPR^40^:

15 $$\begin{gathered} K_{CK} ({\varvec{x}}_{i} ,{\varvec{x}}_{j} ) = K_{SE} ({\varvec{x}}_{i} ,{\varvec{x}}_{j} ) + K_{RQ} ({\varvec{x}}_{i} ,{\varvec{x}}_{j} ) = (\sigma_{f}^{SE} )^{2} \exp [ - \frac{{(x_{i} - x_{j} )^{T} M(x_{i} - x_{j} )}}{2}] + \hfill \\ (\sigma_{f}^{RQ} )^{2} [1 + \frac{{({\varvec{x}}_{i} - {\varvec{x}}_{j} )^{T} M({\varvec{x}}_{i} - {\varvec{x}}_{j} )}}{2\alpha }]^{ - \alpha } + \sigma_{n}^{2} \delta_{ij} \hfill \\ \end{gathered}$$

The improved Gaussian process regression (IGPR) algorithm is a GPR algorithm that uses the CK function shown in Eq. (15).

## PSO-IGPR hybrid algorithm for geomechanical parameter identification

### IGPR model for the non-linear relationship between geomechanical parameters and rock displacement

A non-linear relationship of geomechanical parameters ***X*** (*x*_1_,*x*_2_,…,*x*_n_) with the displacement of the rock mass at key location points ***Y*** (*y*_1_, *y*_2_…, *y*_m_) is represented by $IGPR({\varvec{X}})$ as.

16 $$IGPR({\varvec{X}}):R^{n} \to R^{m}$$

$${\varvec{Y}} = IGPR({\varvec{X}}),\;\user2{Y = }\left( {y_{1} ,\;y_{2} \ldots ,y_{m} } \right),\;\user2{X = }\left( {x_{1} ,\;x_{2} \ldots ,x_{n} } \right).$$

where ***X* = **(*x*_1_,*x*_2_,…,*x*_n_) represents the input variables, including the geomechanical parameters of the rock mass that need to be identified, and ***Y* = **(*y*_1_, *y*_2_…, *y*_m_) is the displacement of key location points.

To obtain IGPR(***X***), a training process based on the known data set is needed. The best way is to train the IGPR algorithm and establish an optimal IGPR model to fit the non-linear relation between geomechanical parameters and displacement of rock mass by using the displacement data and the corresponding geomechanical parameters of rock mass measured in situ. However, such data are generally not available. To solve this problem, numerical simulations of rock masses are usually used. Under the premise of the given geomechanical parameter range of the rock mass, the corresponding rock mass displacements under different geomechanical parameters are obtained by numerical simulations. Numerical simulations in this paper are conducted with FLAC^3D^. The IGPR algorithm is trained with these data, and the intelligent non-linear mapping model IGPR(***X***) between geomechanical parameters and displacements of rock mass can be established. The intelligent IGPR(***X***) model can replace numerical calculations for displacement back analysis.

To establish the IGPR model, the objective function,$g({\varvec{\theta}})$, is defined as follows:

17 $$g({\varvec{\theta}}) = Min\left\{ {\sum\limits_{i = 1}^{s} {FLAC(\user2{X,}d)_{i} } - IGPR({\varvec{\theta}}\left| {\user2{X,}d} \right.)_{i} ]^{2} } \right\}$$

where ${\varvec{\theta}}$ are the hyperparameters of the IGPR algorithm. *FLAC*(***X,**** d*)_*i*_ represents the displacement of the *i*th testing sample calculated by FLAC simulations under the given geomechanical parameters ***X*** of the rock mass and the value of distance* d* between the tunnel face and the monitoring section. $IGPR({\varvec{\theta}}\left| {\user2{X,}d)_{i} } \right.$ indicates the displacement of the *i*th testing sample calculated by the IGPR model under the given geomechanical parameters ***X*** of the rock mass and the value of distance *d* but with unknown hyperparameters ${\varvec{\theta}}$ of the IGPR model. *S* denotes the number of testing samples.

Equation (10) shows that the SE kernel function has three hyperparameters: $\sigma_{f} ,{\mathbf{\ell }}$, and $\sigma_{n}$. As shown in Eq. (11), the RQ kernel function has four hyperparameters: $\sigma_{f} ,{\mathbf{\ell }},\alpha$, and $\sigma_{n}$. The combination kernel function shown in Eq. (15) has 6 hyperparameters: $\sigma_{{_{f} }}^{SE} ,{\mathbf{\ell }}^{SE} ,\sigma_{f}^{RQ} ,{\mathbf{\ell }}^{RQ} ,\alpha$, and $\sigma_{n}$**.** Because the generalization performance of the GPR algorithm is directly affected by the selection of each hyperparameter value, the value of each hyperparameter of GPR must be optimized, which is a typical multiparameter combination optimization problem**.** The conjugate gradient method can be used to obtain the optimal GPR hyperparameters (as shown in Eqs. 13 and 14), but it is challenging to determine the initial value and number of iterations for finding a globally optimal solution.

In recent years, swarm intelligence bionic optimization algorithm has been highly valued and widely used^52,53^. As a bionic global optimization algorithm, the objective function of PSO does not need to be differentiable, and the optimization results do not depend on the initial values and implicit parallel searches. PSO has been widely used to solve multiparameter, multivariable, and multiobjective optimization problems. These advantages of PSO compensate for the shortcomings of the conjugate gradient method. Compared with the genetic algorithm, the PSO algorithm is simpler in theory and easier to implement in programs. In this paper, PSO replaces the conjugate gradient method and automatically searches for the optimal hyperparameters during the training process of the IGPR algorithm to establish the non-linear intelligent mapping model IGPR(***X***) between geomechanical parameters and displacements of rock mass.

### PSO coupled with the IGPR algorithm (PSO-IGPR)

PSO is based on the following model and is used to solve optimization problems^54–56^. In PSO, each solution of the optimization problem is a bird, or “particle”, in the search space. Each particle has a fitness value determined by the optimized function and a speed that determines the direction and distance of flight. The particles will follow the current optimal particle to search the solution space.

In PSO, a group of random particles (random solutions) is initialized. Then, the optimal solution is iteratively determined. In each iteration, particles are updated by tracking two extremes. The first is the optimal solution found by the particle itself. This solution is called the individual extremum *pbest,* and the other extremum is the optimal solution found by the entire population. This extremum is the global extremum *gbest*. After finding these two optimal values, the speed and position of the particle are updated according to the following equations:

18 $$v_{i}^{k + 1} = v_{i}^{k} + c_{1} \times r_{1} \times (pbest_{i}^{k} - P_{i}^{k} ) + c_{2} \times r_{2} \times (gbest^{k} - P_{i}^{k} )$$

19 $$P_{i}^{k + 1} = P_{i}^{k} + v_{i}^{k + 1}$$

where $P_{i}^{k}$ and $P_{i}^{k + 1}$ are the *n*-dimensional vectors that represent the position of the *i*-th particle in the search space at iterations *k* and *k* + 1, respectively;$v_{i}^{k}$ and $v_{i}^{k + 1}$ denote its speed at iterations *k* and *k* + 1, respectively; $pbest_{i}^{k}$ and $gbest^{k}$ are defined as the individual extremum of the *i*-th particle and the global extremum at iteration *k*, respectively; *r*_1_ and *r*_2_ are random numbers between 0 and 1; and *c*_1_ and *c*_2_ represent learning factors. In most cases, *c*_1_ = *c*_2_ = 2.

The individual extremum $pbest_{i}^{k + 1}$ of the *i*-th particle and the global extremum $gbest^{k + 1}$ at iteration *k* + 1 are updated according to the following equation:

20 $$pbest_{i}^{k + 1} = \left\{ {\begin{array}{*{20}c} {P_{i}^{k + 1} ,\quad f(P_{i}^{k + 1} ) < f(pbest_{i}^{k} )} \\ {pbest_{i}^{k} ,\quad f(P_{i}^{k + 1} ) \ge f(pbest_{i}^{k} )} \\ \end{array} } \right.$$

21 $$gbest^{k + 1} = \arg \;\min [f(gbest^{k} ),f(pbest_{i}^{k + 1} )]$$

where $f$ represents the fitness function of the PSO algorithm.

IGPR training is a process of continual readjustment between the hyper-parameters to reduce the prediction error of the IGPR model to a pre-set minimum or stop at a pre-set iteration step. Then, the testing samples are input to the trained IGPR model, and the forecasting results are obtained.

There, we use the PSO algorithm, which is characterized by fast global optimization, to search for the optimal hyper-parameters of the IGPR model and avoid trial calculations. The steps involved in implementing the PSO and IGPR (PSO-IGPR) coupled algorithm are as follows.

1. Initialization of PSO network parameters. The particle population size, number of iterations, initial particle velocities and initial locations of particles are determined. Each particle vector represents an IGPR model. Different models correspond to different parameters of the IGPR algorithm.
2. The IGPR algorithm learns knowledge from the learning samples and predicts the displacement value of each testing sample. Then, the PSO algorithm calculates the individual fitness value *f*_*i*_ of each particle.
3. Compare the fitness *f*_*i*_ calculated in step (2) with the best fitness *f*(*pbest*_*i*_) of the particle in the iteration history. If *f*(*pbest*_*i*_) > *f*_*i*_, *f*_*i*_ is used to replace *f*(*pbest*_*i*_) from the previous round, and new particles replace the particles from the previous round.
4. Compare the best fitness *f*(*pbest*_*i*_) of each particle with the best fitness *f*(*gbest*) of all the particles. If *f*(*pbest*_*i*_) < *f*(*gbest*), then replace the original global best fitness value *f*(*gbest*) with the best fitness value *f*(*pbest*_*i*_) of the particle and save the current state of the particle.
5. When the calculation reaches the pre-set number of iterations, the calculation is finished, and the particle with the minimum fitness value is returned to obtain the optimal solution. Otherwise, a new round of iterations is needed to update the positions and speeds of the particles and generate new particles. Then, the process returns to step (2) until the maximum number of iterations is reached and the calculation is finished.

**Figure 1:**
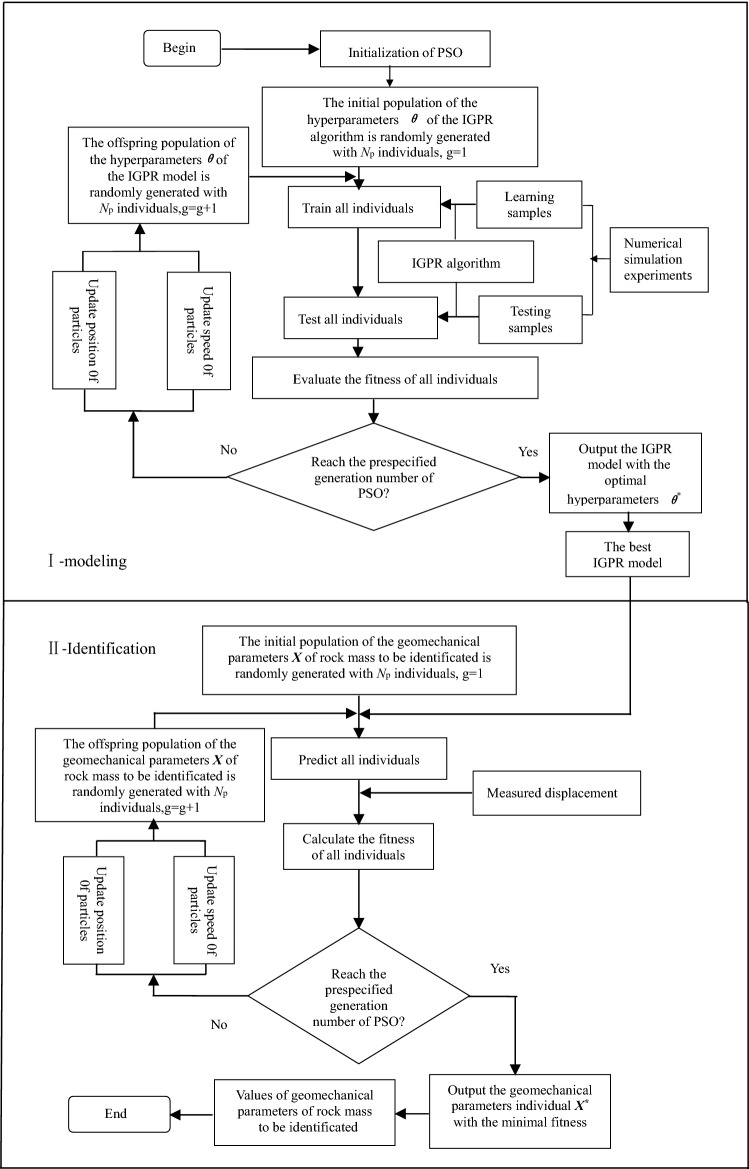
Whole flow chart of direct optimized displacement back analysis based on PSO-IGPR hybrid algorithm.

Figure 1 shows the whole flow chart of the back-analysis of displacement based on the PSO-IGPR hybrid algorithm. Part I of the flow chart represents the modelling stage, i.e., the establishment stage of an IGPR intelligent model that can best fit the non-linear relationship between geomechanical parameters and displacement of rock mass. As shown in Fig. 1, the calculation steps of this stage are as follows:

**Step 1**: Different geomechanical parameters of the rock mass are used for numerical simulation of tunnel construction. The displacements of key points under different geomechanical parameters and different distances between the tunnel face and the monitoring section are recorded, which are used as the training samples of the IGPR algorithm. The training samples $({\varvec{X}}_{i} ,{\varvec{Y}}_{i} )(i = 1,2, \cdots ,k;{\varvec{X}} \in R^{n} ,{\varvec{Y}} \in R^{m} )$ are divided into two parts: the learning samples for the IGPR algorithm to obtain knowledge and the testing samples used to examine the generalizability of the IGPR model.

**Step 2**: The PSO is initialized, and the initial population of the hyperparameters ${\varvec{\theta}}$ of the IGPR algorithm is randomly generated with *N*_p_ individuals. Each individual in the population determines a set of hyperparameters ${\varvec{\theta}}$ of the IGPR algorithm, that is, an IGPR model; the generation counter *g* is set to 1.

**Step 3**: The IGPR algorithm reads $({\varvec{X}}_{i} ,{\varvec{Y}}_{i} )(i = 1,2, \cdots ,k - s;{\varvec{X}} \in R^{n} ,{\varvec{Y}} \in R^{m} )$ in the learning samples and the input variables ${\varvec{X}}_{i} (i = k - 1 + s, \cdots ,k)$ of the testing samples. The hyperparameters ${\varvec{\theta}}$ of each IGPR model in the initial population are read, and then, the learning samples are trained by the IGPR algorithm to obtain knowledge and predict the testing samples.

**Step 4**: The prediction result of each individual of the testing samples is delivered to the following fitness function of the PSO to calculate the fitness of each individual.

22 $$f({\varvec{\theta}}) = \exp \left\{ {0.05 * \arg \min \left[ {\frac{{|IGPR({\varvec{\theta}}\left| {\varvec{X}} \right.,d)_{i} - FLAC(\user2{X,}d)_{i} |}}{{FLAC(\user2{X,}d)_{i} }} \times 100\% } \right]_{i}^{s} } \right\}$$

where $\arg \min [\tfrac{{\left| {IGPR({\varvec{\theta}}\left| {\user2{X,}d)_{i} - FLAC(\user2{X,}d)_{i} } \right.} \right|}}{{FLAC(\user2{X,}d)_{i} }} \times 100\% ]_{i}^{s}$ indicates the minimal relative error between the IGPR model calculation displacement and the FLAC^3D^ calculation displacement of the *s* testing samples.

According to Eq. (22), $f({\varvec{\theta}}) \ge 1$. As the fitness value $f({\varvec{\theta}})$ decreases, the calculated results of the IGPR model more closely approach the simulated values by FLAC. When $f({\varvec{\theta}}) = 1$, the calculated results of the IGPR model are equal to the simulated values by FLAC.

**Step 5**: Compare the fitness *f*_*i*_ calculated in previous step with the best fitness *f*(*pbest*_*i*_) of the particle in the iteration history. If *f*(*pbest*_*i*_) > *f*_*i*_, *f*_*i*_ is used to replace *f*(*pbest*_*i*_) from the previous round, and new particles replace the particles from the previous round. Then, compare the best fitness *f*(*pbest*_*i*_) of each particle with the best fitness *f*(*gbest*) of all the particles. If *f*(*pbest*_*i*_) < *f*(*gbest*), then replace the original global best fitness value *f*(*gbest*) with the best fitness value *f*(*pbest*_*i*_) of the particle and save the current state of the particle.

**Step 6**: Whether the prespecified number of evolution generations of the PSO was reached is determined. If reached, then the calculation stops and the individual IGPR model with the minimal fitness $f({\varvec{\theta}}^{*} )$ is returned; the optimal hyperparameters ${\varvec{\theta}}^{*}$ of the IGPR model have been obtained. Otherwise, a new round of iterations is needed to update the positions and speeds of the particles and generate new particles. Then, the process returns to step 3 until the maximum number of iterations is reached and the calculation is finished. So far, the optimal hyperparameters ${\varvec{\theta}}^{*}$ of the IGPR model have been obtained.

To date, the best non-linear IGPR intelligent model with the optimal hyperparameters ${\varvec{\theta}}^{*}$ mapping the relationship between geomechanical parameters and displacement of rock mass has been established. We can use this best IGPR model to replace the traditional 3D numerical simulation to carry out displacement back analysis, which can greatly reduce the calculation time to meet the construction needs.

### Back analysis for identifying geomechanical parameters of a rock mass using PSO-IGPR

The best IGPR intelligent model with optimal hyperparameters ${\varvec{\theta}}^{*}$ that can map the nonlinear relationship between the geomechanical parameters and the displacement of the rock mass is established in Sect. PSO coupled with the IGPR algorithm (PSO‑IGPR). For intelligent displacement back analysis, the objective function of the geomechanical parameter identification stage for the rock mass is shown in the following equation:

23 $$g({\varvec{X}}) = Min\{ \sum\limits_{i = 1}^{p} {[u_{i} } - IGPR({\varvec{X}}\left| {{\varvec{\theta}}^{ * } \user2{,}d)_{i} } \right.]^{2} \}$$

where $u_{i}$ represents the measured displacement values of the *i*th monitoring point (crown subsidence or horizontal convergence) and $IGPR({\varvec{X}}\left| {{\varvec{\theta}}^{ * } \user2{,}d)} \right._{i}$ indicates the displacement values of the *i*th monitoring point calculated by the best IGPR model under the optimal hyperparameters ${\varvec{\theta}}^{ * }$ and the value of distance *d* but with unknown geomechanical parameters ***X*** of the rock mass. In addition, *p* denotes the number of monitoring points (*p* = 1 here).

As shown in Eq. (23), iteration optimization is used in the proposed displacement back analysis method to search the optimal geomechanical parameters ***X*** of the rock mass by using PSO, which can ensure that the displacement calculated by the best IGPR model is as close to the measured displacement as possible; these parameters are considered the “real geomechanical parameters” of the rock mass, thus completing parameter identification.

Part II of Fig. 1 represents the identification stage, i.e., the identification stage of geomechanical parameters of rock mass based on PSO-IGPR hybrid algorithm. The calculation steps of this stage are as follows:

**Step 1**: The PSO step is initialized, and then, the initial population of the geomechanical parameters ***X*** of the rock mass is randomly generated with *N*_p_ individuals. Each individual in the population determines a set of geomechanical parameters ***X*** of the rock mass; the generation counter *g* is set to 1.

**Step 2**: The best IGPR model reads in each rock mass geomechanics parameter individual of the initial population and the specified distance *d* between the tunnel face and the monitoring section. The key point displacements can be predicted by the excellent generalization performance of the IGPR algorithm.

**Step 3**: The PSO step reads in the corresponding measured displacements of each key point, calculates the fitness values of each rock mass geomechanical parameter according to the fitness function defined below, and evaluates the individual fitness of each rock mass geomechanical parameter.

24 $$f({\varvec{X}}) = \exp \left\{ {0.05 * \arg \min \left[ {\frac{{|u_{i} - IGPR({\varvec{X}}\left| {{\varvec{\theta}}^{ * } \user2{,}d} \right.)_{i} |}}{{u_{i} }} \times 100\% } \right]_{i}^{p} } \right\}$$

where $\arg \min [\tfrac{{\left| {u_{i} - IGPR({\varvec{X}}\left| {{\varvec{\theta}}^{ * } \user2{,}d)_{i} } \right.} \right|}}{{u_{i} }} \times 100\% ]_{i}^{p}$ indicates the minimal relative error between the displacements predicted by the best IGPR model and the measured displacements of the *p* monitoring points.

According to Eq. (24), $f({\varvec{X}}) \ge 1$. The smaller the fitness value is, the closer the displacements predicted by the best IGPR model are to the measured displacements. When $f({\varvec{X}}) = 1$, the calculated displacements of the best IGPR model are equal to the measured displacements.

**Step 4**: Is the prespecified generation number of the PSO reached? If reached, then the calculation stops and the geomechanical parameter individual X^*^ with the minimal fitness $f({\varvec{X}}^{*} )$ is returned; the optimal geomechanical parameters ${\varvec{X}}^{*}$ of the rock mass have been obtained. If not, the calculation procedure proceeds to the next step.

**Step 5**: Perform similar operations similar to steps 5 and 6 of the above training process until the prespecified number of evolution generations of the PSO is reached and the optimal particle individual (*G*_best_) is returned, which is the geomechanical parameter identification value of rock mass.

The geomechanical parameter identification of rock masses based on the PSO-IGPR hybrid algorithm has been completed. Generally, this method consists of two stages:

**STAGE 1**: This stage is the modelling stage, i.e., training the IGPR algorithm with samples obtained from numerical simulation. In this training process, the PSO algorithm is used to automatically search the optimal IGPR hyperparameters ${\varvec{\theta}}^{ * }$, which can bring the displacement predicted by the IGPR algorithm closest to the displacement calculated by numerical simulation, and to establish the IGPR model, which can best fit the non-linear relationship between geomechanical parameters and the displacement of the rock mass.

**STAGE 2**: The second stage is the identification stage. That is, PSO is used to automatically search the optimal geomechanical parameters X^*^ within the geomechanical parameter range of the rock mass, which can bring the displacement predicted by the best IGPR model established in the previous stage closest to the measured displacement, and complete the geomechanical parameter identification of the rock mass.

## Case study on Beikou tunnel

### Brief introduction of the Beikou tunnel

**Figure 2:**
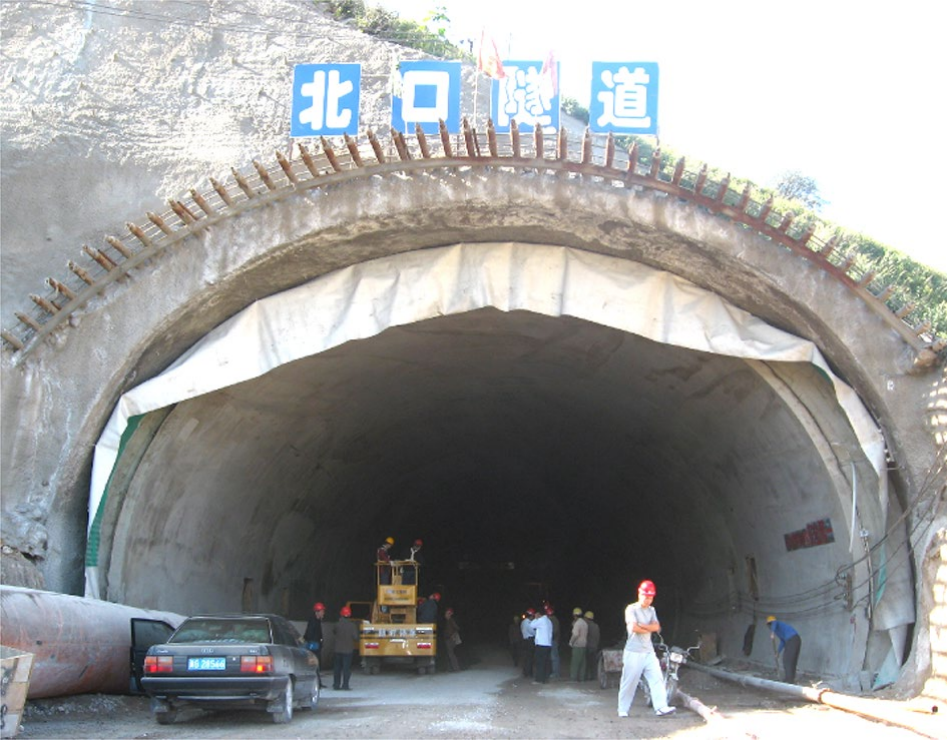
Left entrance of the Beikou tunnel.

**Figure 3:**
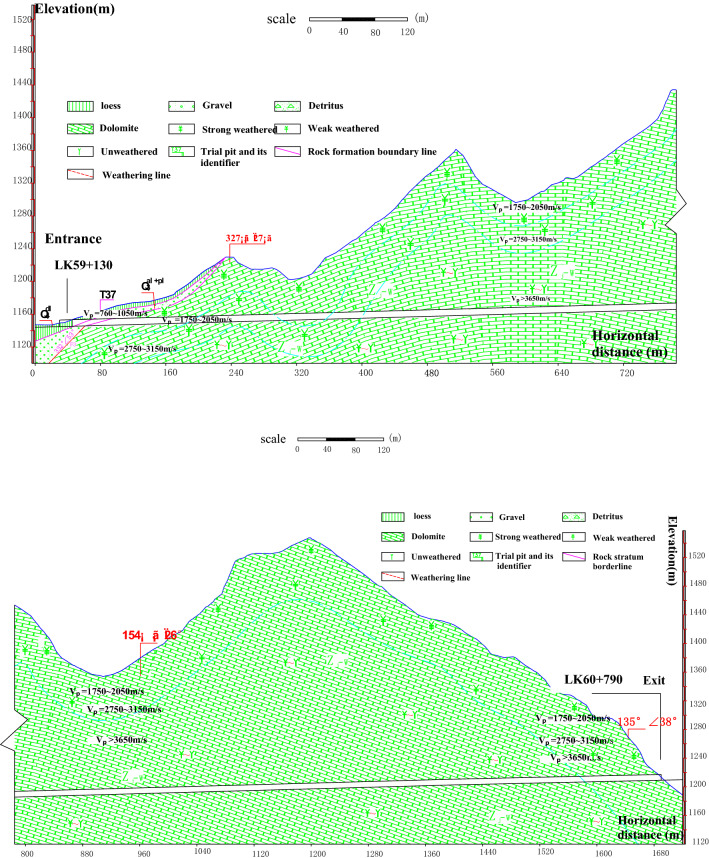
Geological profile of the left line of the Beikou tunnel.

The Beikou tunnel, located in Baoding City, Hebei Province, China, is a twin four-lane separated tunnel that is approximately 1600 m in length. The main geomorphic units crossed by the tunnel are the Sanggan River basin, the low mountains and hilly areas of the Weixian basin, and the medium mountainous areas in southern Weixian County. The tunnel site is a medium–low mountain landform with steep cliffs and developed valleys. The ground elevation of the tunnel is between 1100 m and 1556 m, with a relative elevation of approximately 456 m and a maximum buried depth of approximately 343 m. The main geological structure is an anticline that runs from the tunnel entrance to the mileage pile K60 + 000. The strike of this anticline is NE 61°, and the angle between the tunnel axis and this anticline is approximately 47°. The inclination and dip angle of the north wing rock are NW 327° and 27°–43°, respectively. The inclination and dip angle of the south wing rock are SE 165° and 26°–35°, respectively. The rock mass of the anticline shaft is profoundly broken. There are no active geological structures in the tunneling region. The dolomite joints with high dip angles are relatively developed. The entrance and exit of the tunnel are extremely steep with many vertical fissures; thus, there is a risk of collapse. The left entrance and the geological profile of the left line are shown in Figs. 2 and 3, respectively.

The quality rating result of the rock masses around the tunnel based on the basic quality (BQ) classification method of China is shown in Table 1 based on the initial geological survey.

**Table 1:** BQ classification results of the surrounding rock of the Beikou tunnel.

Position	Mileage	Tunnel length	Cumulative length (m)				
			V grade shallow buried	V grade deep buried	IV grade	III grade	Open cut part
Right line	TRK59 + 240 − 60 + 840	1600	222	35	205	1115	23
Left line	TLK59 + 120 − T60 + 785	1665	156	145	145	1195	24

### IGPR model of rock mass deformation around the Beikou Tunnel

#### Three-dimensional numerical simulations

According to the flow chart described in the previous section, first, numerical simulations should be used to form samples for IGPR training. In this paper, a numerical tool, FLAC3D, is used to carry out 3D numerical simulations to obtain the deformation of rock masses in Beikou tunnel construction.

The chainage numbers at the entrance of the Beikou tunnel for the left and right lines are LK59 + 130 and LK59 + 255, respectively. The entrances of the left and right lines are 125 m apart in the direction of excavation. In addition, the distance from the axis of the left tunnel to the axis of the right tunnel is approximately 71 m, and the maximum excavation width of the tunnel is approximately 11 m. Therefore, the construction of the left tunnel has limited influence on the right tunnel and vice versa. This study modelled only the construction of the left line to shorten the computation time for numerical simulations.

**Figure 4:**
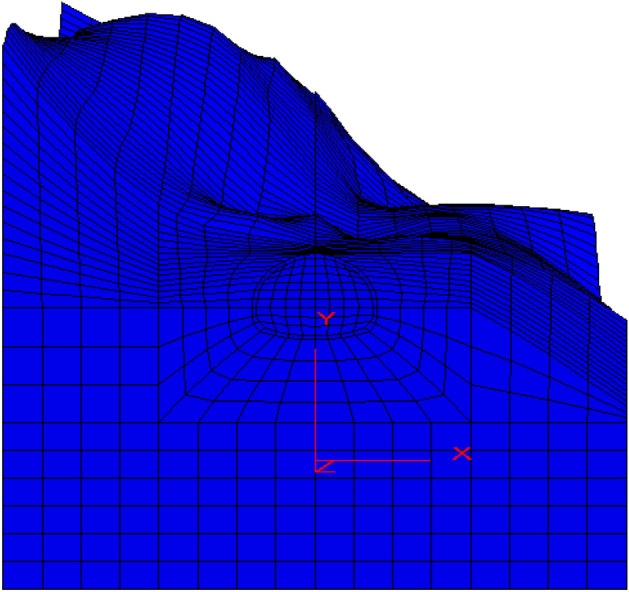
Numerical calculation model of the left entrance of the Beikou tunnel.

The tunnel plane contour map is demarcated, the local coordinate system is set, and the calculation area is meshed. Then, the 3D coordinates of each grid point are read according to the position of the grid point in the local coordinate system and the elevation of the contour line. The grid point coordinates are input into ANSYS software to establish the finite element mesh of the underlying surface hills. Then, the 3D finite element model of the tunnel numerical simulation is established according to the height of the calculation model, and the node and element data of the model established by ANSYS software are transformed into the file format needed for FLAC software modelling using special data format conversion software. Finally, the FLAC3D software reads the data of the format file and directly generates a 3D numerical calculation model, as shown in Fig. 4. The distance from the left boundary to the right boundary of the model is 73.44 m, the length along the excavation direction is 60 m, and the vertical direction is 40.33 m from the crown of the tunnel to the lower boundary. The upper boundary is built according to the actual elevation of the mountain surface. The model consists of 21,520 elements and 24,540 nodes.

The elastoplastic constitutive model and the Mohr**–**Coulomb strength criterion are used for numerical simulation. The normal deformation of each boundary of the model is limited except for that of the Earth’s surface. Because there are no in situ stress data, the ground stress in the vertical direction is taken as the weight of the overlying strata, and the horizontal stress in two directions is assumed to be equal to the vertical stress multiplied by the lateral pressure coefficient.

The excavation was performed by the upper and lower bench excavation method; the excavation of the lower bench of the tunnel face was divided into two parts: left and right. Compared to the right part of the lower bench, the excavation footage of the left part was 15 m ahead. Compared to the left part of the lower bench, the excavation footage of the upper bench was 15 m ahead. Each cyclic excavation was 1.5 m. In this method, preliminary bracing was used for the tunnel face. The initial support parameters of the tunnel excavation are shown in Table 2. The cable structural element and the shell element in the FLAC software are used to simulate the anchor bolt and primary shotcrete, respectively.

**Table 2:** Parameters of the primary support.

Thickness of sprayed concrete	Strength grade of concrete	Bolt type	Bolt length	Bolt diameter	Bolt spacing	Reinforcing mat diameter	Reinforcing mat spacing
cm			m	mm	(cm × cm)	mm	(cm × cm)
15	C25	Hollow grouting anchor	3.0	25	100 × 100	6	20 × 20

Thirty test schemes are designed using the uniform test method for the mechanical parameters of the rock mass and the lateral pressure coefficient. The construction of the left line entrance is simulated by FLAC3D software. The distances *d* between the tunnel face and monitoring section LK59 + 150 are 6, 7.5, and 9 m, respectively. The calculated results are extracted randomly to compose 36 learning samples and 5 testing samples for training the IGPR algorithm, as shown in Table 3.

**Table 3:** Training sample set.

Sample classification	*E *(GPa)	*C *(MPa)	*Φ *(°)	*λ*	*μ*	*d *(m)	Horizontal convergence (mm)	Crown settlement (mm)
Learning samples	18.4	0.85	48	1.2	0.268	6	0.27	0.07
	6.4	1.3	43	1.75	0.254	6	1.21	0.13
	7.6	0.15	42	0.6	0.296	6	0.49	0.76
	2.2	0.4	36	1.05	0.275	6	1.86	0.66
	19	0.45	32	1.45	0.331	6	0.36	0.06
	12.4	0.25	44	1.1	0.443	6	0.32	0.14
	16.6	0.3	27	0.65	0.38	6	0.1	0.32
	5.8	0.8	33	0.8	0.45	6	0.35	0.32
	11.8	1.35	29	1.7	0.436	6	0.6	0.1
	1.6	0.7	39	1.6	0.345	6	4.02	0.27
	17.8	1.25	24	1	0.401	6	0.2	0.09
	13	0.65	30	0.85	0.247	6	0.26	0.15
	8.8	1.5	35	1.35	0.373	7.5	0.67	0.19
	2.8	1.15	46	1.4	0.415	7.5	2.02	0.5
	14.2	0.75	22	1.65	0.303	7.5	0.57	0.07
	8.2	0.5	21	1.25	0.422	7.5	0.63	0.29
	11.2	1.2	40	0.95	0.31	7.5	0.35	0.17
	15.4	1.45	45	0.75	0.338	7.5	0.17	0.14
	16.6	0.3	27	0.65	0.38	7.5	0.11	0.36
	5.8	0.8	33	0.8	0.45	7.5	0.36	0.39
	11.8	1.35	29	1.7	0.436	7.5	0.64	0.11
	1.6	0.7	39	1.6	0.345	7.5	4.29	0.22
	17.8	1.25	24	1	0.401	7.5	0.21	0.12
	13	0.65	30	0.85	0.247	7.5	0.27	0.17
	4.6	1.4	28	1.15	0.289	9	1.12	0.35
	11.8	1.35	29	1.7	0.436	9	0.67	0.13
	19	0.45	32	1.45	0.331	9	0.38	0.07
	2.8	1.15	46	1.4	0.415	9	2.1	0.56
	8.8	1.5	35	1.35	0.373	9	0.69	0.22
	2.2	0.4	36	1.05	0.275	9	2.01	0.74
	11.2	1.2	40	0.95	0.31	9	0.36	0.19
	13.6	1	50	1.55	0.387	9	0.54	0.13
	5.8	0.8	33	0.8	0.45	9	0.36	0.45
	10	0.95	37	0.55	0.408	9	0.07	0.3
	17.8	1.25	24	1	0.401	9	0.22	0.13
	3.4	1.05	23	0.7	0.324	9	0.66	0.7
Testing samples	13.6	1	50	1.55	0.387	6	0.48	0.1
	4.6	1.4	28	1.15	0.289	6	1.02	0.27
	3.4	1.05	23	0.7	0.324	7.5	0.67	0.62
	6.4	1.3	43	1.75	0.254	9	1.37	0.16
	5.2	0.55	49	0.9	0.366	9	0.65	0.41

Here and elsewhere, the symbol “*d*” represents the distance from the tunnel face to monitoring section LK59 + 150.

#### IGPR model for mapping geomechanical parameters to displacement of rock mass

By training on the samples shown in Table 3, PSO is used to search for the optimal hyperparameters ${\varvec{\theta}}^{ * }$ of the IGPR model that can make the displacement calculated by the IGPR model closest to the displacement calculated by the FLAC3D simulations during the sample training process. Therefore, the best IGPR intelligent model with the optimal hyperparameters ${\varvec{\theta}}^{ * }$ that maps the nonlinear relation between the geomechanical parameters and the displacement of the rock mass is established.

Three different algorithms with the same inversion process are used to execute the displacement back analysis for comparison purposes. These algorithms are the PSO and standard GPR coupled (PSO**-**GPR) algorithm with a single kernel function, the PSO-IGPR algorithm and the PSO and SVR coupled (PSO-SVR) algorithm. The PSO parameters of the above three identification methods are identical. The CK function shown in Eq. (15) is used as the kernel function of the IGPR model. The search intervals of $\sigma_{f}$, $\ell$, $\alpha$, and $\sigma_{n}$ are [0,100], [0, 10], [0, 10] and [0, 0.05], respectively. The kernel function of the SVR algorithm is the polynomial kernel function shown below:

25 $$K({\varvec{x}}_{i} ,{\varvec{x}}_{j} ) = ({\varvec{x}}_{i} \cdot {\varvec{x}}_{j} + 1)^{r} ,r = 1,2, \cdots$$

where *r* is a positive integer that represents the order of the polynomial. There are two other parameters of the SVR algorithm: the penalty parameter *C* and fitting error $\varepsilon$^56,57^. The search intervals of SVR parameters $C$ and $\varepsilon$ are [0, 5000] and [0, 1], respectively, and the kernel parameter *k* is 1, 2 or 3.

The population size of the PSO is 20, and the number of evolution generations is 500.

The output variable of the standard GPR and standard SVR algorithms can only be a one-dimensional scalar^32,33^; therefore, two intelligent models mapping the relationship of the crown subsidence and the horizontal convergence to the geomechanical parameters of the rock mass are established.

Rasmussen^34^ developed the open source program of the GPR algorithm based on MATLAB language. On this basis, the code of the PSO-IGPR hybrid algorithm is programmed in MATLAB according to the flowchart shown in Fig. 1 and Eqs. (22) and (24) and then applied to the training sample set shown in Table 3. The best GPR, IGPR and SVR models with the optimal hyperparameters and parameters searched by the PSO on the basis of the steps described in Section “PSO coupled with the IGPR algorithm (PSO-IGPR)” are shown in Tables 4, 5 and 6, respectively.

**Table 4:** Optimal hyperparameters of the best GPR model.

Hyper parameter value	$\sigma_{f}^{{}}$	$l_{1}$	$l_{2}$	$l_{3}$	$l_{4}$	$l_{5}$	$l_{6}$	$\alpha$	σ_*n*_
**Kernel function**									
Horizontal convergence									
SEard	1.79	4.90	9.09	4.64	0.68	4.15	6.69		0.05
RQard	2.29	0.21	7.30	0	5.83	6.58	4.51	1.13	0.01
SEiso	1.67	0.37							0.03
RQiso	1.68	0.64						2.95	0.04
Crown subsidence									
SEard	9.99	1.50	6.69	1.52	3.84	7.98	8.02		0.05
RQard	1.20	9.99	8.57	0.30	0.08	1.87	0.97	3.54	0.01
SEiso	1.34	9.41							0.04
RQiso	1.34	9.97						10.0	0

**Table 5:** Optimal hyperparameters of the best IGPR model.

Optimal hyper parameter value	SEard							RQard								σ_n_
	$\sigma_{f}^{{}}$	$l_{1}$	$l_{2}$	$l_{3}$	$l_{4}$	$l_{5}$	$l_{6}$	$\sigma_{f}^{{}}$	$l_{1}$	$l_{2}$	$l_{3}$	$l_{4}$	$l_{5}$	$l_{6}$	α	
**Deformation of surrounding rock**																
Horizontal convergence	9.45	0.31	1.52	8.96	4.09	4.27	3.15	1.17	8.09	7.9	8.65	0.2	7.99	4.69	9.18	0.02
Crown subsidence	7.21	8.81	1.66	9.57	8.51	3.28	6.36	1.49	8.25	7.04	0.55	1.42	4.47	6.02	9.75	0.05

Optimal hyper parameter value	SEiso		RQiso			σ_n_										
	$\sigma_{f}^{{}}$	*l*	$\sigma_{f}^{{}}$	*l*	α											
**Deformation of surrounding rock**																
Horizontal convergence	2.88	1.4	1.71	1.61	0.14	0.04										
Crown subsidence	41.05	6.99	10.79	9.68	8.69	0.04

**Table 6:** Optimal parameters of the best SVR model.

Parameters	Horizontal convergence			Crown subsidence		
	*C*	*r*	*ε*	*C*	*r*	*ε*
**Kernel function**						
Poly	1898.4	1	0	99.97	1	0.01

### Rock mass parameter identification for the Beikou tunnel using PSO

The geomechanical parameters of the rock mass to be identified are the deformation modulus *E*, cohesion *C*, internal friction angle *Φ*, horizontal lateral pressure coefficient λ and Poisson's ratio μ. According to the value range of the geomechanical parameters of the different grades of rock mass specified in the *Standard for engineering classification of rock masses*^58^, the search intervals of the geomechanical parameters are provided in Table 7.

**Table 7:** Searching intervals of the geomechanical parameters to be identified.

Parameter	*E *(GPa)	*C *(MPa)	*Φ *(°)	*λ*	*μ*
Interval	1.0 ~ 19	0.05 ~ 1.5	21 ~ 50	0.55 ~ 2	0.45 ~ 0.25

The best intelligent GPR, IGPR and SVR models (Tables 4, 5 and 6) that can map the nonlinear relationship between the displacement and geomechanical parameters of the rock mass around the Beikou tunnel are established in Section “IGPR model of rock mass deformation around the Beikou tunnel”. The measured horizontal convergence and crown subsidence values are input into these intelligent models. The distance between the tunnel face and monitoring section LK59 + 150 is 6 m. The number of evolution generations and the population size of the PSO are 500 and 20, respectively. The other parameters of the PSO remain unchanged. The fitness function of the PSO is adopted the Eq. (24) during the geomechanical parameter identification stage.

The identification results obtained using the PSO are shown in Table 8.

**Table 8:** Geomechanical parameters identified by the displacement of the rock mass around the tunnel of section LK59 + 150 (*d* = 6.0 m).

Identification value of parameters	*E *(GPa)	*C *(MPa)	*Φ *(°)	*λ*	*μ*
**Identification algorithm**					
Horizontal convergence					
PSO-GPR(SEard)	9.8	0.83	29.69	1.62	0.26
PSO-GPR(RQard)	11.03	0.7	35.11	1.66	0.4
PSO-IGPR(CKard)	13.47	0.72	47.91	1.59	0.34
PSO-SVR	13.14	1.35	47.51	1.63	0.35
PSO-GPR(SEiso)	10.43	0.89	30.21	1.83	0.44
PSO-GPR(RQiso)	11.96	1.13	23.03	1.76	0.3
PSO-IGPR(CKiso)	1.88	1.39	31.61	0.61	0.41
Crown subsidence					
PSO-GPR(SEard)	4.23	0.44	29.96	0.8	0.36
PSO-GPR(RQard)	3.17	1.42	30.28	1.06	0.27
PSO-IGPR(CKard)	4.45	0.40	48.06	0.61	0.26
PSO-SVR	2.42	0.56	28.33	0.57	0.28
PSO-GPR(SEiso)	4.24	1.41	24.28	1.22	0.33
PSO-GPR(RQiso)	2.31	0.18	45.61	0.68	0.36
PSO-IGPR(CKiso)	2.1	0.8	39.98	0.76	0.41

Then, the identified parameter values shown in Table 8 are input into the intelligent models shown in Tables 4, 5 and 6; the horizontal convergence and crown subsidence values in monitoring section LK59 + 150 can be forecast for the following excavation. The results are shown in Tables 9 and 10.

**Table 9:** Horizontal convergence values forecasted by the identified geomechanical parameters of monitoring section LK59 + 150.

*D *(m)	Measured horizontal convergence (mm)	Horizontal convergence values forecasted by the GPR algorithm (mm)						SVR forecast horizontal convergence (mm)	GPR forecast relative error (%)						SVR forecast relative error (%)
		GPR				IGPR			GPR				IGPR		
		SE		RQ		CK			SE		RQ		CK		
		ard	iso	ard	iso	ard	iso		ard	iso	ard	iso	ard	iso	
7.5	0.73	0.75	0.73	0.74	0.71	0.73	0.72	0.69	2.74	0	1.37	2.74	0	1.37	5.48
9	0.78	0.8	0.76	0.8	0.73	0.78	0.81	0.72	2.56	2.56	2.56	6.41	0	3.85	7.69
10.5	0.9	0.82	0.77	0.87	0.76	0.84	0.93	0.74	8.89	14.44	3.33	15.56	6.66	3.33	17.78
12	0.94	0.82	0.78	0.94	0.78	0.89	1.05	0.77	12.77	17.02	0	17.02	5.32	11.7	18.09
13.5	1.05	0.83	0.78	1.0	0.79	0.95	1.17	0.79	22.86	25.71	4.76	24.76	9.52	11.43	24.76
Average relative error (%)									9.96	11.95	2.4	13.3	4.3	6.34	14.76

**Table 10:** Crown subsidence values forecasted by the identified geomechanical parameters of monitoring section LK59 + 150.

*d* (m)	Measured crown subsidence (mm)	Crown subsidence values forecasted by the GPR algorithm (mm)						SVR forecast crown subsidence (mm)	GPR forecast relative error (%)						SVR forecast relative error (%)
		GPR				IGPR			GPR				IGPR		
		SE		RQ		CK			SE		RQ		CK		
		ard	iso	ard	iso	ard	iso		ard	iso	ard	iso	ard	iso	
7.5	0.63	0.54	0.53	0.55	0.56	0.6	0.58	0.52	14.29	15.87	12.7	11.11	4.76	7.94	17.46
9	0.77	0.58	0.56	0.61	0.58	0.71	0.61	0.55	24.68	27.27	20.78	24.68	7.79	20.78	28.57
10.5	0.92	0.62	0.58	0.66	0.61	0.82	0.65	0.57	32.61	36.96	28.26	33.7	10.87	29.35	38.04
12	1.02	0.66	0.61	0.7	0.65	0.95	0.68	0.6	35.29	40.2	31.37	36.27	6.86	33.33	41.18
13.5	1.17	0.71	0.62	0.71	0.69	1.09	0.72	0.62	39.32	47.01	39.32	41.03	6.84	38.46	47.01
Average relative error (%)									29.24	33.46	26.49	29.36	7.42	25.97	34.45

### Analysis of calculation results

Table 9 illustrates that the maximum prediction error of GPR is slightly larger than that of SVR, but the average relative error is less than that of the SVR algorithm when using identification parameters to predict the surrounding rock deformation for five successive excavation steps. This result means that all of the GPR algorithms have higher identification accuracy than the SVR algorithm. A similar result can be obtained from the crown subsidence results forecasted using the identified parameters for subsequent excavation in Table 10. Overall, the prediction precisions of the three types of GPR algorithms are better than that of the SVR algorithm regardless of whether an ARD-type or ISO-type kernel function is used. Moreover, the GPR algorithm with an ARD-type kernel function is better than the GPR algorithm with an ISO-type kernel function for the same form of the kernel. When the same type of kernel function is used, the IGPR algorithm has higher identification accuracy than the GPR algorithm with a single kernel function. The SVR algorithm has the lowest identification accuracy.

The prediction accuracy of the IGPR algorithm with an ARD-type combination kernel function is exceptionally high. The maximal prediction relative error of the surrounding rock deformation using the identification parameters for five successive excavation steps is only 10.26%; the average prediction relative errors of the horizontal convergence and the crown subsidence are 4.3% and 7.39%, respectively, which fully satisfies the needs of tunnel construction. For the IGPR algorithm with an ISO-type combination kernel function, the maximal prediction relative error of the surrounding rock deformation using the identification parameters for five successive excavation steps is 38.46%, and the average prediction relative errors of the horizontal convergence and crown subsidence are 6.34% and 25.97%, respectively. Therefore, the IGPR algorithm with an ARD-type combination kernel function has higher identification accuracy than the IGPR algorithm with an ISO-type combination kernel function.

### Discussion

#### Robustness of CKGPR model

All three machine learning algorithms, namely, SVR, standard GPR and IGPR, are comparatively presented briefly for intelligent displacement back analysis in this paper. In contrast, SVR does not perform as well as standard GPR and IGPR under less raw data conditions. Since GPR derives the mapping from probability theory with prior and covariance functions, the prediction of GPR is a probabilistic distribution; GPR can apply the mean of the distribution as point predictions to avoid robust point predictions like that in SVR, which leads to GPR performing slightly better than SVR in the practical application of displacement back analysis.

In order to test the robustness of the IGPR (ARD type) model established in this paper, the second test sample in Table 3 is taken as the model input benchmark sample and adopted the super parameters of IGPR model shown in Table 5. The input parameters of this sample are changed step by step, the corresponding prediction output of the model is checked, and compared with the sample output value (benchmark value) to observe the robustness of the model. The change range of each input parameter is increased or decreased by 10%, the calculation results are shown in the following table:

It can be seen from the calculation results in Table 11 that when the input parameters of IGPR model increase by 10% one by one, compared with the benchmark value, the maximum relative error of horizontal convergence prediction of the model is 6.5%, and the average relative error of prediction is only 4.03%; The maximum relative error of the model is 11.5%, and the average relative error is only 5.12% When the input parameters of IGPR model are reduced by 10% one by one, compared with the benchmark value, the maximum relative error of horizontal convergence prediction of the model is 16.8%, and the average relative error of prediction is only 5.13%; The maximum relative error of the model is 15.4%, and the average relative error is only 8.96% It can be seen that the ard type igpr model established in this paper has good robustness.

**Table 11:** Model robustness detection and analysis table.

Model input parameters						Model prediction output	Relative error of model prediction (%)	Model prediction output	Relative error of model prediction (%)
*E* (GPa)	*C* (MPa)	*Φ* (°)	*λ*	*μ*	*d* (m)	Horizontal convergence (mm)		Crown subsidence (mm)	
**Input parameters are increased by 10% one by one**									
4.6	1.4	28	1.15	0.289	6	1.07		0.26	
5.06	1.4	28	1.15	0.289	6	1.14	6.50	0.26	0
4.6	1.54	28	1.15	0.289	6	1.13	5.60	0.26	0
4.6	1.4	30.8	1.15	0.289	6	1.14	6.50	0.29	11.5
4.6	1.4	28	1.27	0.289	6	1.07	0.00	0.25	3.85
4.6	1.4	28	1.15	0.318	6	1.02	4.67	0.24	7.7
4.6	1.4	28	1.15	0.289	6.6	1.08	0.93	0.28	7.7
**Input parameters are reduced by 10% one by one**									
4.6	1.4	28	1.15	0.289	6	1.07		0.26	
4.14	1.4	28	1.15	0.289	6	1.08	0.93	0.29	11.5
4.6	1.26	28	1.15	0.289	6	1	6.50	0.26	0
4.6	1.4	25.2	1.15	0.289	6	0.89	16.80	0.23	11.5
4.6	1.4	28	1.04	0.289	6	1.07	0.00	0.3	15.4
4.6	1.4	28	1.15	0.26	6	1.13	5.61	0.28	7.7
4.6	1.4	28	1.15	0.289	5.4	1.06	0.93	0.24	7.7

#### Computational efficiency analysis

For the GPR algorithm, the relevant measure hyperparameter ℓ of the ARD-type kernel function is more than that of the ISO-type kernel function. Therefore, the generalization performance of the GPR algorithm increases with an increasing number of parameters. By comparison, the number of hyperparameters of the IGPR algorithm with an ARD-type combination kernel function is the largest, and its generalization performance is the best among the three algorithms.

Computation time is monitored by defining a clock function in the program; the computation times for different algorithms in the sample training and parameter identification phases are shown in Table 12 (personal computer configuration for a dual-core processor, 3.1 GB and 4 GB of memory).

**Table 12:** Comparison of the calculation times during different stages (unit: s).

Algorithm		GPR						SVR
Kernel function		SE		RQ		CK		Poly
Kernel function type		ard	iso	ard	iso	ard	iso	
Sample training	Horizontal convergence	49.699	47.861	64.473	51.467	96.648	73.351	230.112
	Crown subsidence	49.655	47.795	65.236	53.53	98.357	81.037	257.345
Parameter identification	Horizontal convergence	46.573	46.535	64.320	47.684	87.299	73.939	377.35
	Crown subsidence	47.555	47.541	66.993	49.724	89.782	75.068	381.592

Table 12 demonstrates that in the sample training stage, the computation time of the SVR algorithm is approximately 2–5 times that of GPR; in the parameter identification stage, the computation time of SVR is approximately 4–8 times that of GPR. The GPR algorithm is far more efficient than the SVR algorithm.

#### Limitations and future studies

For multi-parameter direct optimization inversion, the biggest problem is the non-uniqueness of the solution, which brings great difficulties to practical application. The feasible solution is to limit the search interval of each parameter to be inversed as much as possible, that is, to enhance the priori of the parameters, which is the next research focus of multi-parameter displacement back analysis.

The amount of data is a critical factor that affects the analysis results for any machine learning algorithm. With limited data, it is difficult to obtain an accurate and robust model. Therefore, the trained model should be used carefully. In addition, the prediction result of the learning machine also depends on the quality of the data and its generalization performance, which is directly affected by the value of the parameters. In this paper, PSO is a coupled learning machine to automatically search for the best parameters of the learning machine in the training process, and the effect is excellent. However, it is necessary to further study the parameter selection theory of the GP.

## Conclusions

The following conclusions are obtained through the above comparative study:

1. The GPR algorithm can be used to identify the geomechanical parameters of rock masses. This can improve the accuracy of the identification compared to that when using the SVR algorithm.
2. The IGPR algorithm can improve the generalization performance and the identification precision of the GPR algorithm with a single kernel function. The average relative prediction error of the IGPR algorithm with an ARD-type kernel function is approximately 27**–**74% lower than that of the GPR algorithm with a single kernel function; that of the IGPR algorithm with an ISO-type kernel function is approximately 11**–**52% lower than that of the GPR algorithm with a single kernel function. In comparison, the ARD-type combination kernel GPR has the largest number of hyperparameters, but the identification accuracy is significantly improved without a considerable increase in computation time.
3. The maximal relative error of each excavation step and the maximal average relative error of all excavation steps forecasted by the IGPR algorithm based on the ARD-type kernel function are only 10.26% and 7.39%, respectively, over five excavation steps. This result shows that the novel 3D displacement back analysis method based on the PSO**-**IPGR hybrid algorithm with the ARD-type kernel function proposed in this paper has very high accuracy when identifying the geomechanical parameters of the rock mass and can be applied for parameter identification in geotechnical engineering applications.

## Method

The geomechanical parameters of rock mass are the key parameters directly related to the stress and strain calculation of rock mass engineering, and the geomechanical parameters of rock mass are usually "inaccurate" whether in indoor experiment or in-situ test. Therefore, the displacement back analysis method is usually used to identify the geomechanics of rock mass in engineering. In order to meet the needs of construction, artificial intelligence technology is widely used in displacement back analysis to identify the geomechanical parameters of rock mass. Aiming at the shortcomings of existing artificial neural network (ANN) and support vector regression (SVR) in the application of three-dimensional displacement back analysis, Gaussian process regression (GPR) algorithm is introduced to make up for the shortcomings of existing intelligent identification methods. In order to improve the generalization performance of single kernel GPR algorithm, two single kernel functions are added to form a combined kernel function. An improved Gaussian process regression (IGPR) algorithm based on combined kernel function is proposed. In addition, in the training process of IGPR model, in order to quickly find the super parameters of IGPR model with the best training effect, particle swarm optimization (PSO) and IGPR model are combined to automatically search the parameters of IGPR model to form PSO-IGPR coupling model. FLAC3D software is used for three-dimensional numerical simulation of tunnel engineering construction to form a training sample set with rock mass geomechanical parameters and the distance between tunnel face and monitoring section as input parameters, and the calculated crown subsidence and horizontal convergence as output parameters, which is used for the training of IGPR model. The training steps of the IGPR model are as follows:

1. *Initialization* of PSO parameters. The particle population size, number of iterations, initial particle velocities and initial locations of particles are determined. Each particle vector represents an IGPR model. Different models correspond to different parameters of the IGPR algorithm.
2. The IGPR algorithm learns knowledge from the learning samples and predicts the displacement value of each testing sample. Then, the PSO algorithm calculates the individual fitness value *f*_*i*_ of each particle.
3. Compare the fitness *f*_*i*_ calculated in step (2) with the best fitness *f*(*pbest*_*i*_) of the particle in the iteration history. If *f*(*pbest*_*i*_) > *f*_*i*_, *f*_*i*_ is used to replace *f*(*pbest*_*i*_) from the previous round, and new particles replace the particles from the previous round.
4. Compare the best fitness *f*(*pbest*_*i*_) of each particle with the best fitness *f*(*gbest*) of all the particles. If *f*(*pbest*_*i*_) < *f*(*gbest*), then replace the original global best fitness value *f*(*gbest*) with the best fitness value *f*(*pbest*_*i*_) of the particle and save the current state of the particle.
5. When the calculation reaches the preset number of evolution generations of PSO, the calculation is finished, and the particle with the minimum fitness value is returned to obtain the optimal solution. Otherwise, a new round of iterations is needed to update the positions and speeds of the particles and generate new particles. Then, the process returns to step (2) until the maximum number of iterations is reached and the calculation is finished.

After the training process is completed, the IGPR model has been able to accurately map the relationship between geomechanical parameters and rock mass deformation, then directly use PSO algorithm to search the best geomechanical parameters of rock mass, so as to make the deformation calculated by IGPR model closest to the measured deformation of rock mass, and complete the identification of rock mass geomechanical parameters. The steps of training process are as follows:

1. The PSO is initialized, and then, the initial population of the geomechanical parameters X of the rock mass is randomly generated with *N*_*p*_ individuals. Each individual in the population determines a set of geomechanical parameters X of the rock mass; the generation counter g is set to 1.
2. The best IGPR model reads in each rock mass geomechanics parameter individual of the initial population and the specified distance d between the tunnel face and the monitoring section. The key point displacements can be predicted by the excellent generalization performance of the IGPR algorithm.
3. The PSO reads in the corresponding measured displacements of each key point, calculates the fitness values of each rock mass geomechanical parameter according to the fitness function of PSO, and evaluates the individual fitness of each rock mass geomechanical parameter.
4. Is the prespecified generation number of the PSO reached? If reached, then the calculation stops and the geomechanical parameter individual with the minimal fitness is returned; the optimal geomechanical parameters of the rock mass have been obtained. If not, the calculation procedure proceeds to the next step.
5. Perform similar operations similar to step 3 to 5 of the above training process until the prespecified number of evolution generations of the PSO is reached and the optimal particle individual (*G*_best_) is returned, which is the geomechanical parameter identification value of rock mass.

The application example of Beikou tunnel shows that the combined kernel function IGPR has higher identification accuracy than the single kernel function GPR and SVR model, the IGPR model with automatic correlation deterministic (ARD) kernel function has higher identification accuracy than the IGPR model with isotropic (ISO) kernel function, and the PSO-IGPR hybrid model based on ARD kernel function has the highest identification accuracy. Therefore, a displacement back analysis method of PSO-IGPR hybrid algorithm based on ARD kernel function is proposed in this paper, which can be used to identify the geomechanical parameters of rock mass and solve other engineering problems.
